# Autophagy modulation altered differentiation capacity of CD146^+^ cells toward endothelial cells, pericytes, and cardiomyocytes

**DOI:** 10.1186/s13287-020-01656-0

**Published:** 2020-03-26

**Authors:** Mehdi Hassanpour, Jafar Rezaie, Masoud Darabi, Amirataollah Hiradfar, Reza Rahbarghazi, Mohammad Nouri

**Affiliations:** 1 Department of Biochemistry and Clinical Laboratories, Faculty of Medicine, Tabriz University of Medical Sciences, Tabriz, Iran; 2 Stem Cell Research Center, Tabriz University of Medical Sciences, Imam Reza St., Golgasht St., Tabriz, 5166614756 Iran; 3 Cardiovascular Research Center, Tabriz University of Medical Sciences, Tabriz, Iran; 4 Solid Tumor Research Center, Cellular and Molecular Medicine Institute, Urmia University of Medical Sciences, Urmia, Iran; 5 Pediatric Health Research Center, Tabriz University of Medical Sciences, Tabriz, Iran; 6 Drug Applied Research Center, Tabriz University of Medical Sciences, Tabriz, Iran

**Keywords:** Human bone marrow CD146^+^ cells, Autophagy modulation, Nitrosative assay, Lineage-specific differentiation, Exosome activity

## Abstract

**Background:**

To date, many attempts are employed to increase the regenerative potential of stem cells. In this study, we evaluated the hypothesis of whether an autophagy modulation could alter differentiation potency of CD146^+^ cells into mature pericyte, endothelial, and cardiomyocyte lineage.

**Methods:**

In this study, CD146^+^cells were enriched from the human bone marrow aspirates and trans-differentiated into mature endothelial cells, pericytes, and cardiomyocytes after exposure to autophagy stimulator (50-μM Met)/inhibitor (15-μM HCQ). The protein levels of autophagy proteins were monitored by western blotting. NO content was measured using the Griess assay. Using real-time PCR assay and western blotting, we monitored the lineage protein and gene levels. Pro-inflammatory cytokine and angiocrine factors were measured by ELISA. The fatty acid change was determined by gas chromatography. We also measured exosome secretion capacity by measuring AChE activity and real-time PCR assay.

**Result:**

Data revealed the modulation of autophagy factors, Beclin-1, P62, and LC3 II/I ratio in differentiating CD146^+^ cells after exposure to Met and HCQ (*p* < 0.05). The inhibition of autophagy increased NO content compared to the Met-treated cells (*p* < 0.05). Real-time PCR analysis showed that the treatment of CD146^+^ cells with autophagy modulators altered the expression of VE-cadherin, cTnI, and α-SMA (*p* < 0.05). Met increased the expression of VE-cadherin, α-SMA, and cTnI compared to the HCQ-treated cells (*p* < 0.05) while western blotting revealed the protein synthesis of all lineage-specific proteins under the stimulation and inhibition of autophagy. None statistically significant differences were found in the levels of Tie-1, Tie-2, VEGFR-1, and VEGFR-2 after autophagy modulation. Fatty acid profile analysis revealed the increase of unsaturated fatty acids after exposure to HCQ (*p* < 0.05). The treatment of cells with HCQ increased the levels of TNF-α and IL-6 compared to the Met-treated cells. Data revealed the increase of exosome biogenesis and secretion to the supernatant in cells treated with HCQ compared to the Met groups (*p* < 0.05).

**Conclusions:**

In summary, autophagy modulation could alter differentiation potency of CD146^+^cells which is important in cardiac regeneration.

## Background

According to WHO statistics, coronary heart disease is the main cause of human mortality in the clinical setting. Despite current advances in the decrease mortality rate in patients with coronary heart disease, further attempts and novel approaches are highly needed for the alleviation of cardiac tissue injuries [1]. Up to date, the advent of cell therapy and regenerative medicine is touted as the modern therapeutic procedures in the field of cardiovascular disease [2]. Hitherto, different types of stem cells, including embryonic stem cells, cardiac progenitor cells, mesenchymal stem cells, endothelial progenitor cells, and induced pluripotent stem cells have been used in the clinical setting. Recently, it has been documented that PCs have great regenerative potential in limb ischemia and myocardial infarction [3, 4]. PCs are a specific population that concentrically wraps vascular ECs [5]. According to recent data, a fraction of PCs exhibits extreme proliferation, and clonogenic capacity with a magnificent stemness feature, pleiotropic properties, and angiogenic potency [6]. Based on molecular analyses, multiple PC populations have the potential to express OCT4, NANOG, SOX2, CD146, alkaline phosphatase, CD44, CD73, CD90, CD105, etc. [7]. The ability of PCs to express CD146 (melanoma cell adhesion molecules) was shown before which contributes to highly proliferative rate differentiation capacity [8]. According to previously published experiments, cardiac tissue encompasses a large number of CD146^+^ cells inside myocardium with a trilineage potential capacity to rescue angiogenesis and replace damaged myocardium [9]. Cardiac PCs are potent to release tissue metalloproteinases to re-model the fibrous matrix in the periphery of the injured sites [10]. These features make PCs as an appropriate cell source for the alleviation of cardiac tissue injuries.

Autophagy is an intricate intracellular process that facilitates the degradation of impaired proteins and the removal of dysfunctional organelles by fusion to lysosomes and releases out of the cell. In addition to the integrity of autophagy to the bioactivity of the host cells, novel data highlighted the interaction and interplay of the autophagy signaling pathway with another signaling cascade reciprocally [11]. Scientific literature demonstrated that autophagy functions in the developmental and maturation of B lymphocytes, adipocytes, osteoblasts, keratinocytes, and erythrocytes [12–16]. Interestingly, it was revealed that autophagy inhibition resulted in ROS accumulation in hematopoietic stem cells and self-renewal capacity loss [17]. Zhang et al. showed that the promotion of autophagy in C-kit^+^cardiac progenitor cells, accelerated differentiation toward mature CMs after inhibition of the fibroblast growth factor signaling pathway, showing the critical role of autophagy in differentiation properties of stem cells [18, 19].

Despite PCs’ significance in hemostasis, angiogenesis, vasculogenesis, differentiation, and maturation into several cardiac cells, there is no information related to the modulation of autophagy on the differentiation potential of immature PCs. In this study, we further evaluated the hypothesis, whether an autophagy modulation could alter differentiation potency of CD146^+^ cells into mature PCs, ECs, and CMs.

## Methods

### Mononuclear cell isolation and expansion protocol

Bone marrow samples of healthy volunteers, ranging from 3 to 10 years old, referred to children hospital, an affiliated hospital to Tabriz University of Medical Sciences, were enrolled in the present study. Informed consent was obtained from the parents/legally authorized representatives of participants. Children who suspected of malignant diseases and clinical screening were referred to routine bone marrow aspiration procedures at the posterior superior iliac crest. In the current experiment, we selected the samples without any neoplastic changes, genetic, and inflammatory diseases. Briefly, bone marrow aspirates (2 ml) were used for the isolation of MNCs. To inhibit the coagulation, a 5000-IU/mL heparin was used. Blood samples were diluted with PBS (1:1 v/v) and overlaid on the same volume of Ficoll-Hypaque solution (Cat No: GE17-1440-02, Sigma-Aldrich). The samples were centrifuged at 400*g* for 25 min at 4 °C. MNCs at the interface fraction between the plasma and the Ficoll solution were carefully collected, washed with PBS, and re-suspended in DMEM/LG (Cat No: 31600083, Gibco, USA) culture medium. The media were supplemented with %10 FBS (Cat No: 10270, Invitrogen) and replaced every 3–4 days. By using 0.25% Trypsin-EDTA (Cat No: 25200056, Gibco, USA) solution, cells were detached.

### Enrichment of CD146^+^ cells using magnetic-activated cell sorting

In the current study, we aimed to isolate CD146^+^ cells for different analyses. For this purpose, expanded bone marrow MNCs were detached using the enzymatic solution and subjected to MACS. In short, the MNCs were blocked by using 1% bovine serum albumin for 20–30 min and incubated with mouse anti-human CD146 microbead (order no: 130-093-596, Miltenyi Biotec, Germany) for 30 min at 4 °C. The cell suspensions were passed over the MACS LS column (order no: 130-042-401, Miltenyi Biotec).

### Cell survival assay

This study aimed to evaluate the effect of autophagy modulation on the differentiation capacity of CD146^+^ cells toward different lineages. In this regard, we performed MTT assay to select the maximum dose of autophagy blocker, HCQ, with the lowest toxic effect on CD146^+^ cells. In this regard, CD146^+^ cells were plated (2 × 10^4^/well) in each well of 96-well plates (SPL). Cells were treated with different concentrations of HCQ (Cat No: H0915, Sigma-Aldrich) including 2.5, 5, 10, 15, and 20 μM for 72 h [20]. Thereafter, a 30-μl MTT solution was added to each well and incubated at 37 °C for 2 h followed by the addition of 200-μl dimethyl sulfoxide (Merck, Germany). The optical density was read at 620 nm by using a microplate reader (BioTek). The cell survival rate was expressed as a percentage relative to the non-treated control CD146^+^ cells. To stimulate autophagy, CD146^+^ cells were treated with a 50-mM Met (as a gift from Osveh Pharmaceutical Inc., Tehran, Iran) [21].

### LysoTracker assay

To assess the inhibitory effect of HCQ on the late stage of autophagy, we performed LysoTracker staining. To this end, MNCs were seeded at a density of 10^4^ cells per well in 8-well Chambered Cell Culture Slide (SPL) and incubated at 37 °C with 5% CO_2_ and 95% relative humidity. After 24 h, cells were treated with 15- and 20-μM HCQ for 72 h. After completion of autophagy modulation, cells were washed with cold PBS, 50 nM LysoTracker Green (cat no: L7526, Sigma-Aldrich) added to each well and kept for 30 min. After three times of washing with PBS, cells were stained with a 1-μg/ml DAPI (Sigma-Aldrich) solution 30 s to stain the background. The cells harboring intracellular vacuoles were visualized by using immunofluorescence microscopy (Model: BX41, Olympus).

### Cell differentiation and autophagy modulation

**Fig. 1:**
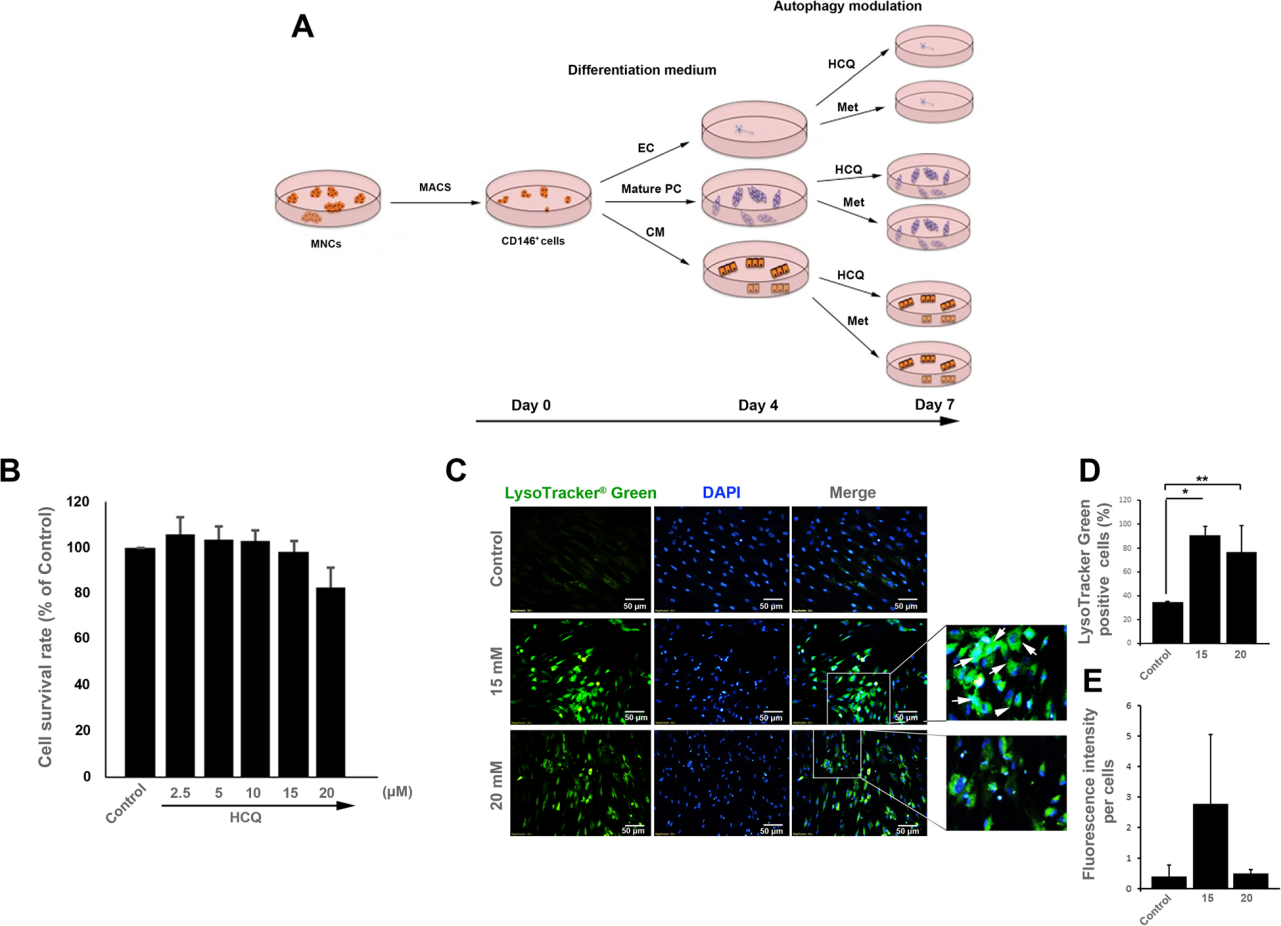
Schematic illustration of study design (**a**). MTT assay (**b**); Measuring CD146^+^ cell survival rate by MTT assay after exposure to different doses of HCQ (*n* = 16). Our data illustrated that 15-μM HCQ is the optimum dose for autophagy inhibition in CD146^+^ cells with a minimal toxic effect. LysoTracker Green staining (**c**–**e**). Fluorescence microscopy imaging confirmed that 15-μM HCQ inhibited autophagic flux (**c**, **d** (*n* = 100 cells) and **e** (*n* = 100 cells)). Data are expressed as mean ± SD. One-way ANOVA and Tukey post hoc test. *p < 0.05; **p < 0.01

In this study, we explored the effect of autophagy modulation on the differentiation potency of CD146^+^ cells in vitro. Purified CD146^+^ cells were cultured in the endothelium (Cat No: C-22111, Promocell, Germany), pericyte (Cat No: C-28040, Promocell, Germany), and cardiomyocyte (Cat No: 05010, STEMCELL, USA) differentiation media. Cells were maintained for 7 days in differentiation media supplemented with 2% FBS and 1% Pen-Strep solutions. On day 4, autophagy was blocked/stimulated using 15-μM HCQ and 50-μM Met (Cat No: Osveh Pharmaceutical Inc., Iran) as previously described (Fig. 1a) [21].

### Western blotting

Western blot analysis was done to evaluate and confirm the modulation of autophagy proteins in PC-, CM-, and EC-like cells after the modulation of autophagy. On day 7, cells were collected and lysed in ice-cold cell lysis buffer solution (NaCl, NP-40, and Tris–HCl) enriched with cocktail enzyme inhibitors. Then, the samples were sonicated at a resonance of 50 Hz for 2 min. The homogenates were centrifuged at 14,000 g for 20 min. By using the Picodrop spectrophotometer system (Model No: PICOPET01, Serial No. 000212/1), total protein content was determined in the supernatant. In this study, 100-μg proteins from each sample were resolved by 12% SDS-PAGE method and transferred to PVDF membrane. The membranes were incubated in antibody solution (LC3: Cat No: ab51520, Abcam; P62: Cat No: SC-10117, Santa Cruz Biotechnology, Inc.; Beclin-1: Cat No: SC-48341, Santa Cruz Biotechnology, Inc.; α-actinin-2: Cat No: SC-130928, Santa Cruz Biotechnology, Inc.; α-SMA: Cat No: SC-53015, Santa Cruz Biotechnology, Inc.; vWF: Cat No: SC-365712, Santa Cruz Biotechnology, Inc.; ERK 1/2: Cat No: SC-292838, Santa Cruz Biotechnology, Inc.; pERK 1/2: Cat No: SC-16981-R, Santa Cruz Biotechnology, Inc.) at 4 °C overnight. Then, the membranes were incubated with secondary HRP-conjugated anti-IgG antibody (Cat No: sc-2357, Santa Cruz Biotechnology, Inc.) for 1 h at room temperature. The immunoreactive blots were detected using the ECL plus solution (BioRad). Immunoreactive bands were analyzed with ImageJ software (ver.1.44p, NIH). GAPDH (Cat No: sc-32233, Santa Cruz Biotechnology, Inc.) was used as an internal control group. This experiment was performed in triplicate.

### NO assay

The level of NO was measured in differentiating cells after autophagy stimulation or inhibition using the Griess method. In this method, NO is converted to nitrite and nitrite produces nitrous acid in an acidic state. In the next step, the addition of sulfanilamide to a solution containing nitrous acid forms a diazonium salt and in the presence of n-(1-naphthyl) ethylenediamine dihydrochloride (C_12_H_16_Cl_2_N_2_) generates an azo dye. Briefly, an initial number of 1 × 10^4^ cells was seeded in each well of 96-well plates (SPL). Seventy-two hours after the addition of autophagy blocker/stimulator under differentiation condition, 200 μl supernatant of each sample was mixed with 20-μl Griess A and maintained for 10 min followed by the addition of 20 μl of Griess B solution. After 2 min, the optical density of each sample was read at 450 nm using a microplate reader (BioTek) and levels of NO calculated based on sodium nitrite standard curve. Levels of NO were expressed as nM.

### Measuring endothelial RTKs and pro-inflammatory cytokines

To determine the levels of inflammatory cytokines and RTKs during CD146^+^ differentiation into different lineages, we measured the levels of IL-6 (Cat No: DY206, R&D) and TNF-α (Cat No: DY210, R&D) in the supernatants by using ELISA according to manufacturer’s recommendation. The angiogenic status of cells was further assessed by monitoring RTK protein levels such as VEGFR-1 (Cat No: ab32152, Abcam), VEGFR-2 (Cat No: ab39256, Abcam), Tie-1 (Cat No: ab27851, Abcam), Tie-2 (Cat No: ab24859, Abcam), and Ang-1 (Cat No: 130-06, Peprotech) by ELISA assay provided by our group [22]. The final absorbance was recorded at 450 nm using a microplate reader (BioTek, USA). The concentration of each factor either in autophagy stimulated/inhibited and groups is calculated based on comparison to corresponding standard curves.

### Measuring fatty acid composition by GC

The lipid profile was analyzed by the GC technique as previously described [23]. The cellular lipid was extracted by using the Bligh-Dyer method and further esterified with methanol during acetyl chloride catalysis [24]. Methyl esters were introduced to the analyzing machine to determine the fatty acid composition. The fatty acid methyl ester derivatives were separated on a Teknokroma TR CN100 column (60 × 0.25 mm) using a Buck Scientific gas chromatograph (Model 610, SRI Instruments, Torrance, USA). Tridecanoic acid (13:0) was exploited as the internal control. Peak retention times in each group were compared to the peak of standards.

### Assessing paracrine potency of CD146^+^ cells under the modulation of autophagy

To this end, we monitored AChE touted as an exosome marker protein. In brief, supernatants were discarded after the completion of the experimental procedure and replaced by incubation with culture medium containing 1% exosome-free FBS (Invitrogen) for 48 h. Thereafter, a 20-μl culture medium was mixed with 500-μl buffer solution containing pyrophosphate (75 mM) and potassium hexacyanoferrate (2 mM) (Cat No: BXC0801, biorexfars) for 5 min at RT. Finally, a 100-μl s-butyrylthiocholine iodide was added to each sample and absorbance read at 405 nm during three different intervals. Finally, the choline esterase activity was calculated by the following formula: activity (U/l) = ∆Abs/min × 65,800.

### RT-PCR assay

After a 7-day incubation with differentiation media and autophagy modulation, the total RNAs were isolated from each sample. To this end, cells were trypsinized and were washed with PBS. Then, a 1-ml Ambion TRIzol buffer (Cat No: 15596-026, Invitrogen, USA) was added to cell pellets and agitated rigidly to homogenize the samples. Next, 200-μl chloroform (Merck, Darmstadt, Germany) was added and samples centrifuged at 4 °C at 12000 rpm for 20 min. Next, the equal volume of the upper phase containing RNA was mixed with isopropanol (Sigma-Aldrich) and incubated at 4 °C for 15 min. After centrifugation, the supernatant was discarded and the pellet was dissolved in 1-ml ethanol (75% v/v) solution. Finally, the total RNA was dissolved in 50-μl DEPC-treated water and samples’ concentration determined by a Nanodrop system (Thermo Scientific). To exclude a possible genomic DNA contamination, RNA solutions were incubated with the DNase1 kit (Cat No: en0521, FermenTaz). Before real-time PCR analysis, purified RNAs were reversely transcribed using a cDNA synthesis kit (Cat No: YT4500). The qRT-PCR reaction was performed by using 1-μl cDNA, 5-μl SYBR premix Ex Taq kit (Cat No: RR820L, TaKaRa), 0.4 μl candidate gene primers, and 3.2-μl DEPC water. The qRT-PCR was performed by a Rotor-Gene Corbett System 6000. The raw data was analyzed by a convenient Pfaffl method with normalization to housekeeping gene β2-microglobulin (β_2_M). The experiment was conducted in triplicate. The sequences of all primers for human VE-cadherin, cTnI, α-SMA, and TPBG were outlined in Table 1.

**Table 1:** The list of primers designed by Perl Primer software

Gene	Forward	Reverse	Tm (°C)
α-SMA	5′-GTCCACCGCAAATGCTTCTA-3′	5′-AAACACATAGGTAACGAGTCAG-3′	57
VE-cadherin	5′-ACCCAAGATGTGGCCTTTAG-3′	5′-GTGACAACAGCGAGGTGTAA-3′	60
Troponin I	5′-TTTGACCTTCGAGGCAAGTTT-3′	5′-CCCGGTTTTCCTTCTCGGTG-3′	57
β2M	5′-AGGCTATCCAGCGTACTCC-3′	5′-ATGTCGGATGGATGAAACCC-3′	58
CD63	5′-TCCTGAGTCAGACCATAATCC-3′	5′-GATGGCAAACGTGATCATAAG-3′	63
ALIX	5′-CTGGAAGGATGCTTTCGATAAAGG-3′	5′-AGGCTGCACAATTGAACAACAC-3′	63
Rab11	5′-CCTCAGCCTCTACGAAGCAAA-3′	5′-CCGGAAGTTGATCTCCTCCTG-3′	59
Rab27a	5′-AGAGGAGGAAGCCATAGCAC-3′	5′-CATGACCATTTGATCGCACCAC-3′	59
Rab27b	5′-GGAACTGGCTGACAAATATGG-3’	5′-CAGTATCAGGGATTTGTGTCTT-3′	59

### Statistical analysis

In this study, data are expressed as mean ± SD. To find statistically significant differences between the groups, we performed a one-way ANOVA test. *P* < 0.05 was considered statistically significant. Three sets of experiments were at least performed for different analyses unless mentioned.

## Results

### HCQ dose selection for the subsequent analyses

The optimum dosage of HCQ to inhibit autophagy was calculated based on the MTT assay. CD146^+^ cells were exposed to different doses of HCQ ranging from 2.5 to 20 μM for 3 days (Fig. 1b). According to our data, a 3-day incubation of CD146^+^ cells with 2.5-, 5-, and 10-μM HCQ had no cytotoxic effect on cell survival rate. Data demonstrated that a slight increase in the cell survival rate from groups 2.5-, 5-, and 10-μM HCQ. The addition of 15- and 20-μM HCQ to the culture medium decreased cell viability to 98.3 and 82.7%, respectively, showing dose-dependent cytotoxicity of HCQ on the CD146^+^ cells (Fig. 1b). Consistent with the previous experiments, we selected 15-μM HCQ as the highest dose without toxic effect for other analyses [25].

### HCQ treatment showed lysosomal accumulation inside the CD146^+^ cells

To further confirm the suppression of autophagy in CD146^+^ cells, the LysoTracker staining was done (Fig. 1c–e). According to results, the fluorescence intensity and the number of LysoTracker^+^ cells were increased in cells from 15-μM HCQ compared to the control non-treated cells (*p* < 0.05, Fig. 1c–e). We also found that the number of cells with accumulated lysosomes was increased in the 20-μM HCQ group compared to the control cells (*p* < 0.05, Fig. 1c–e). However, these effects were less compared to the 15-μM HCQ. We found a non-significant difference between groups 15- and 20-μM HCQ. These data showed that a 3-day incubation of CD146^+^ cells with HCQ at doses 15- and 20-μM HCQ could efficiently block autophagy efflux and accumulate lysosomes inside the CD146^+^ stem cells. According to data from MTT and IF assays, we selected 15-μM HCQ for subsequent analyses.

### HCQ and Met changed protein levels of autophagy-related factors

**Fig. 2:**
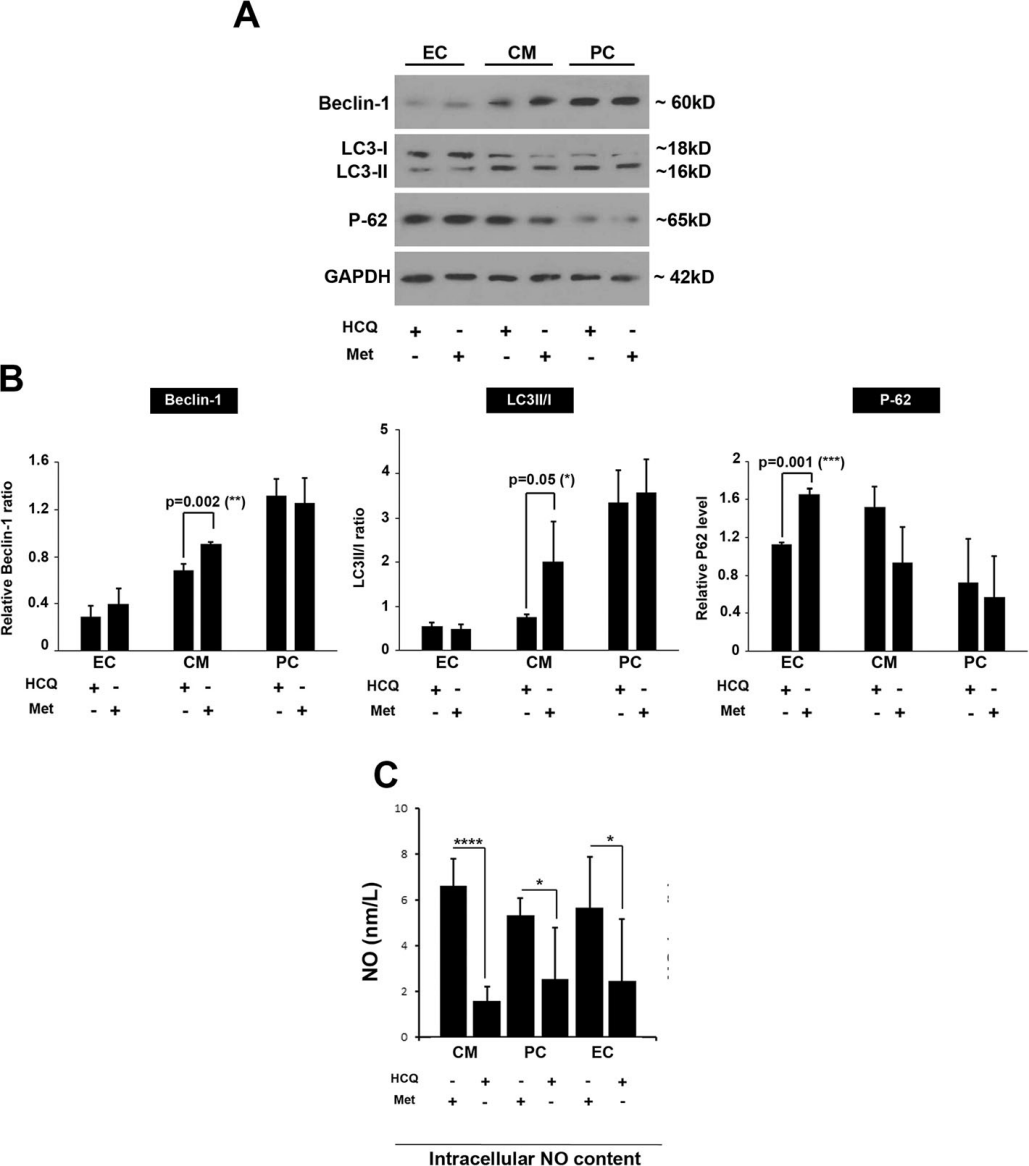
Western blotting and NO assay (**a**–**c**). Measuring protein levels of autophagy-related factors by western blotting (**a**, **b**; *n* = 3). Detecting NO levels by Griess assay (**c**; *n* = 16). Data are expressed as mean ± SD. One-way ANOVA and Tukey post hoc test. *p < 0.05; ****p < 0.0001

Following the completion of the experimental protocol, we performed western blotting to measure protein levels of Beclin-1, P62, and LC3II/I ratio (Fig. 2a, b). We showed that the basal levels of each autophagy factor were different in CD146^+^ cells after being-exposed to different differentiation media. The basal level of Beclin-1 was low in CD146^+^ cells differentiating toward endothelial lineage compared to the other groups. In support of this notion, we found that the P62 was also low in the PC group compared to the cells exposed to CM and EC differentiation media (Fig. 2a, b). According to our data, the treatment of CD146^+^ cells with the Met and endothelial differentiation medium significantly increased protein levels of P62 compared to the group treated with the HCQ group (*p* < 0.05). No statistically significant differences were found between the two groups for the Beclin-1 level and LC3II/I ratio (*p* > 0.05, Fig. 2a, b). The incubation of CD146^+^ cells with Met and CM differentiation medium increased Beclin-1 and LC3II/I ratio compared to the HCQ-treated cells (*p* < 0.05). However, the levels of P62 were decreased in Met-treated cells but did not reach statistically significant levels. We found a statistically non-significant difference in the protein levels of Beclin-1, P62, and LC3II/I ratio in CD146^+^ cells incubated with PC differentiation medium enriched with Met or HCQ (Fig. 2a, b). According to our data, one could hypothesize that the application of autophagy modulators (Met and HCQ) in CD146^+^ cells committed to different lineages changes the protein levels of autophagy-related proteins differently, showing possibly different basal levels of autophagy function in different lineages.

### NO production was increased after autophagy stimulation

To monitor the level of NO production in differentiation cells subjected to autophagy modulation, we performed a Griess assay. Results indicated that NO content was diminished by autophagy suppression via 15-μM HCQ compared to control-matched Met groups after incubation with CM, PC, and EC differentiation medium (*p* < 0.05, Fig. 2c). Overall, NO production was significantly hindered by autophagy inhibition while the stimulation of autophagy increases the NO production especially in CD146^+^ cells exposed to PC and EC differentiation media, showing NO production depends on the cell lineage.

### HCQ and Met altered levels of differentiation-related proteins

**Fig. 3:**
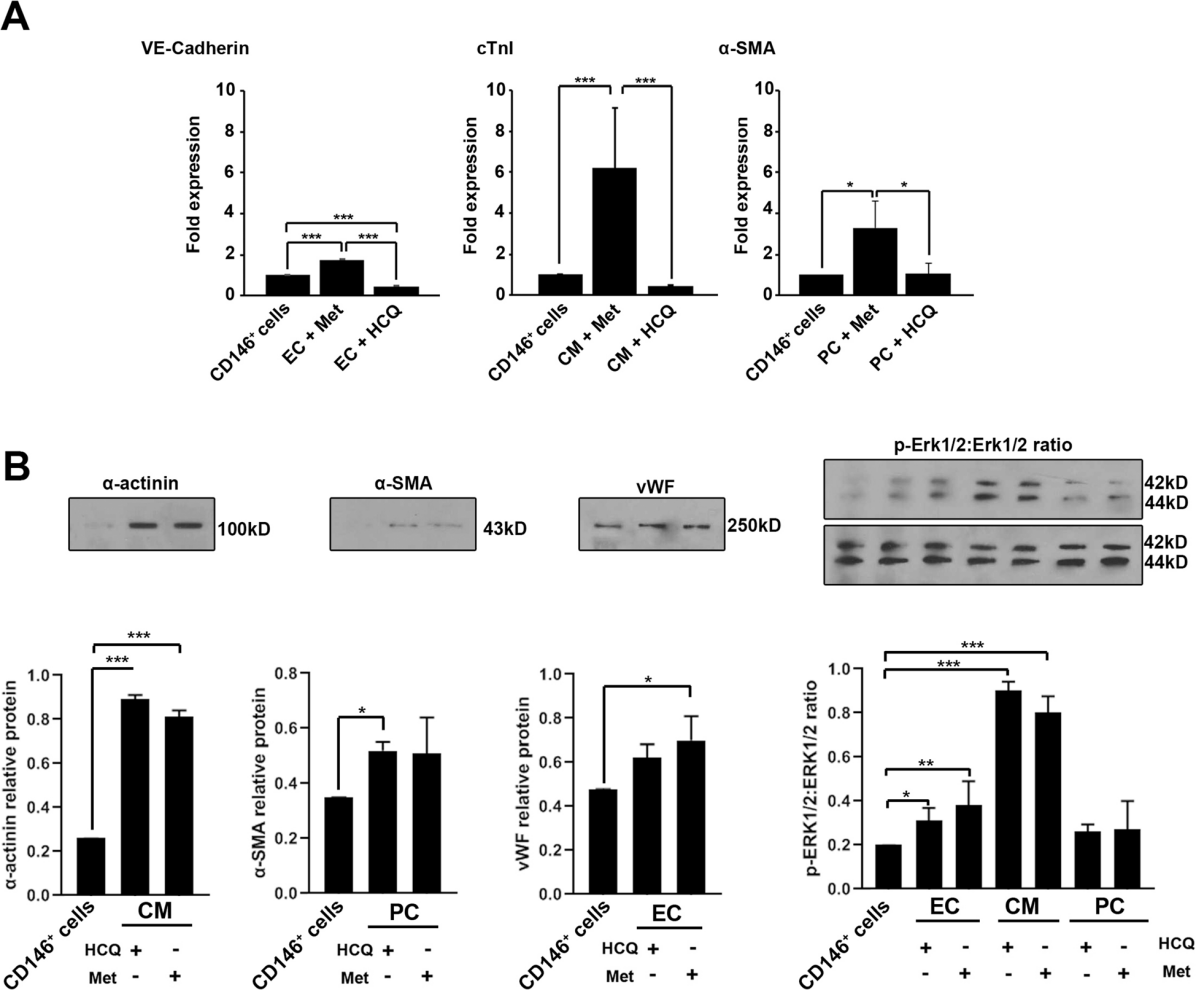
Real-time PCR assay and western blotting (**a**, **b**). Measuring the effect of autophagy modulation on the differentiation capacity of human bone marrow CD146^+^ cells via real-time PCR assay (**a**; *n* = 3). Data demonstrated that autophagy stimulation increased the mRNA level of endothelial VE-cadherin, cardiac cTnI, and pericyte α-SMA after autophagy stimulation compared to non-treated CD146^+^cells (**a**). Western blotting assays were used to monitor lineage protein levels after autophagy modulation (**b**; *n* = 3). One-way ANOVA and Tukey post hoc test. *p < 0.05; **p < 0.01; ***p < 0.001; and ****p < 0.0001

We performed western blotting and real-time PCR analysis to assess the differentiation potential of CD146^+^ cells toward different lineages (Fig. 3a, b). Real-time PCR analysis showed that the treatment of CD146^+^ cells with autophagy modulators altered the expression of VE-cadherin, cTnI, and α-SMA. According to our data, the incubation of CD146^+^ cells with Met significantly increased the expression of VE-cadherin, cTnI, and α-SMA genes compared to the control and HCQ groups (*p* < 0.05, Fig. 3a). We found a significant inhibition of VE-cadherin in the group received HCQ compared to the control CD146^+^ cells (*p* < 0.001, Fig. 3a) while non-significant differences were found in the mRNA levels of both cTnI and α-SMA between the HCQ and control groups. These data demonstrated that the stimulation of autophagy signaling in CD146^+^ cells upon treatment with differentiation medium could increase differentiation potential toward multiple lineages. Western blotting showed that the modulation of CD146^+^ cells with autophagy modulators (either Met or HCQ) could promote lineage-specific protein synthesis compared to the control of CD146^+^ cells (*p* < 0.05, Fig. 3b). The protein analysis of VE-cadherin showed a significant increase in differentiating cells that committed endothelial lineage after exposure to the HCQ and Met. Of note, protein levels of α-SMA and vWF increased in CD146^+^ cells committed to PC and CM differentiation upon treatment with Met and HCQ. The levels of α-SMA and vWF reached significant levels in the differentiation medium enriched with Met and HCQ, respectively (Fig. 3b). Data revealed the significant phosphorylation of Erk1/2 in all cells exposed to differentiation media upon autophagy stimulation and inhibition (*p* < 0.05, Fig. 3b). Despite a slight increase in Erk1/2 phosphorylation of CD146^+^ cells exposed to PC lineage, these changes did not reach statistically significant differences.

### Autophagy modulation effect on RTK levels and pro-inflammatory cytokines

**Fig. 4:**
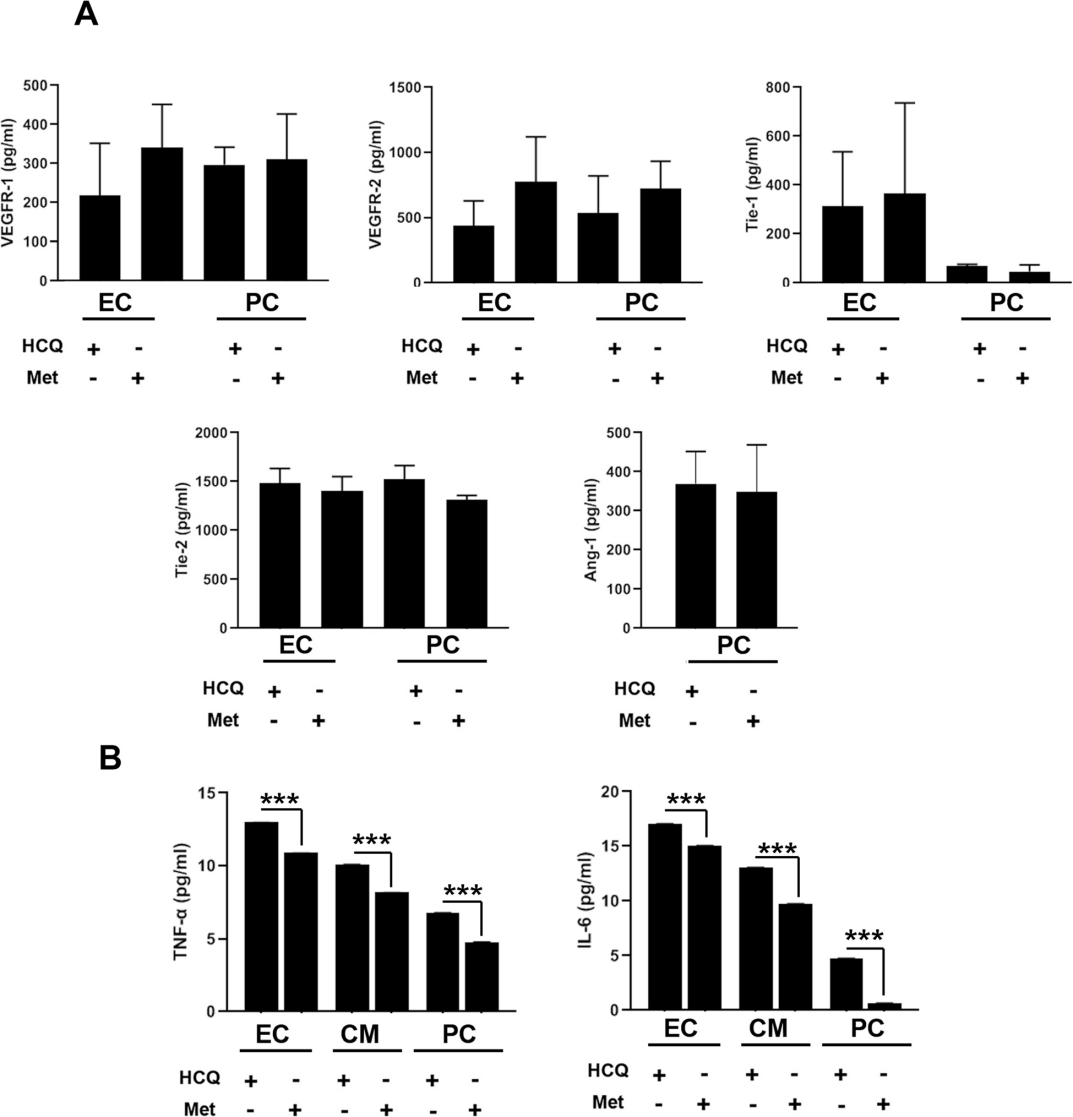
ELISA assay (**a**, **b**). Measuring protein levels of VEGFR-1, VEGFR-2, and Tie-1, Tie-2, and Ang-1 after autophagy modulation (**a**; *n* = 3). It seems that autophagy stimulation could increase differentiation potential, but these changes did not reach statistically significant levels. The effect of autophagy stimulation/inhibition on the inflammatory status of differentiating CD146^+^ cells (**b**; *n* = 3). It seems that autophagy stimulation could decrease significantly the production of TNF-α and IL-6 after treatment with 50-μM Met. Student *t* test. ***p < 0.001

Cell surface RTK levels, VEGFR-1, VEGFR-2, Tie-1, Tie-2, and ligand Ang-1 were monitored by using ELISA. We found an increase in protein level for Tie-1, Tie-2, VEGFR-1, and VEGFR-2 under autophagy stimulation compared to the condition cells received HCQ (Fig. 4a). Despite slight changes, the protein levels of RTKs and Ang-1 did not reach statistically significant differences. Also, it seems that autophagy modulation for 72 h could not alter the potency of CD146^+^ cells to synthesize Ang-1 (Fig. 4a). To the assessment of the correlation between autophagy and the inflammatory process, we measured TNF-α and IL-6 (Fig. 4b). Data showed that autophagy induction by 50-μM Met diminished either TNF-α or IL-6 levels in CD146^+^ cells incubated in EC and PC and CM differentiation media compared to the control-matched group exposed to the 15-μM HCQ (Fig. 4b). According to data, we found that the CD146^+^ cells had a magnificent TNF-α and IL-6 synthesis capacity when committed to the endothelial lineage rather than PC and CM destination (Fig. 4b). Data showed that the promotion of autophagy by Met, although it did not, changes the RTKs and Ang-1 levels but could affect pro-inflammatory status via the reduction of TNF-α and IL-6 cytokines.

### Fatty acid profile was changed during the autophagy modulation in differentiating CD146^+^ cells

**Fig. 5:**
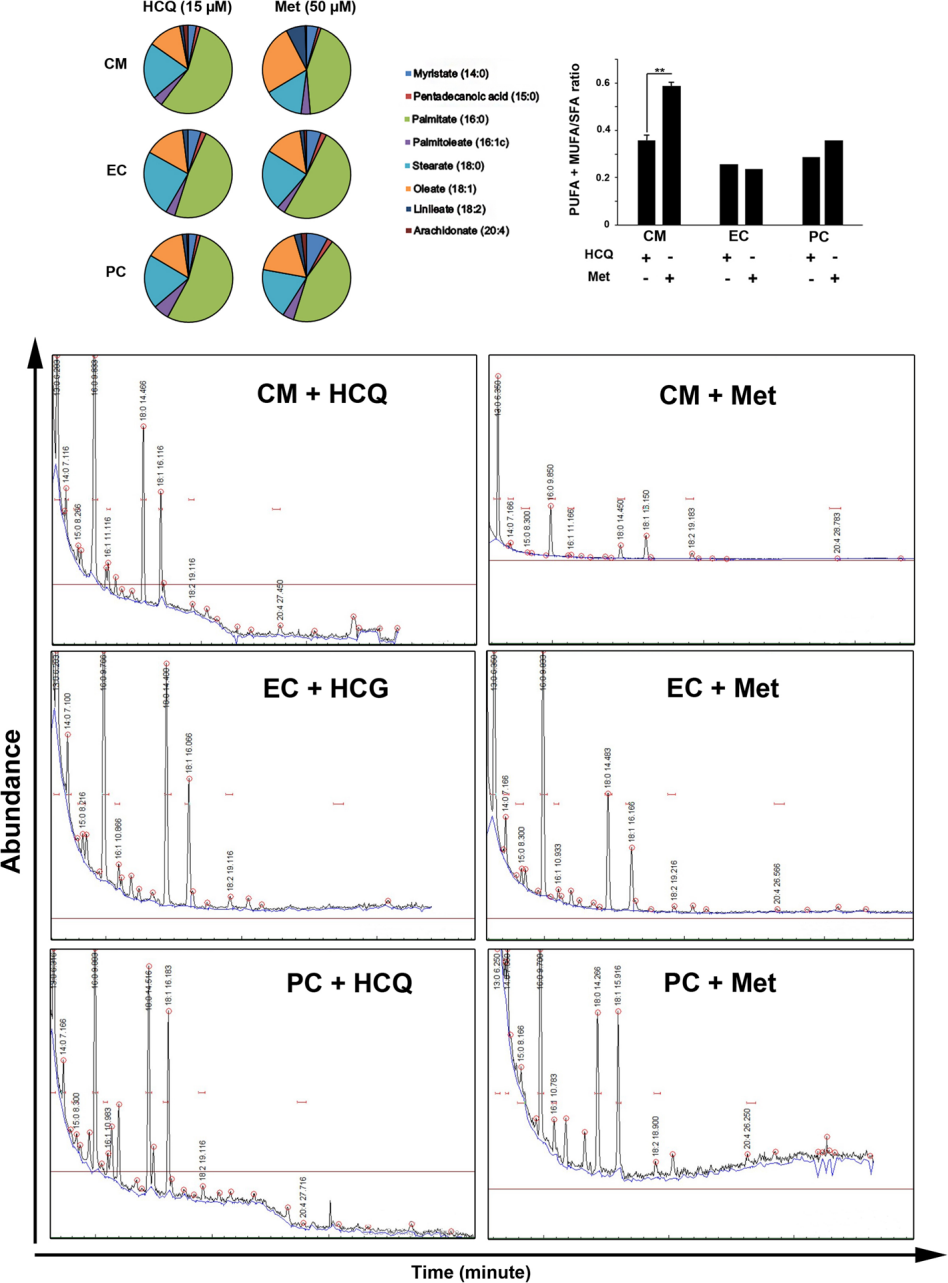
Fatty acid analysis. Gas chromatography fatty acid analysis indicated that the ratio of PUFA + MUFA/SFA was increased after autophagy stimulation in the CM group (*n* = 3). Student *t* test. **p < 0.01

In the current experiment, we measured the level of PUFA (Linoleate18:2 and Arachidonate 20:4), MUFA (Palmitoleate 16:1, Oleate 18:1), and SFA acids (Myristate 14:0, Pentadecanoic acid 15:0 and Palmitate 16:0 and Stearate 18:0) by GC analysis to monitor the ratio of unsaturated and saturate fatty acids in CD146^+^ cells exposed to autophagy stimulation and inhibition (Fig. 5). Data showed the increase of PUFA +MUFA: SFA ratio in CD146^+^ cells after the inhibition of autophagy by HCQ. We found a 2.53-fold increase in the fatty acid ratio in the CM group exposed to 50-μM Met compared to HCQ-treated CM during differentiation (*p* < 0.05, Fig. 5). Similar to the CM group, a 1.24-fold increase was found in the PUFA + MUFA/SFA ratio in PC after exposure to the Met in comparison with the HCQ. In contrast, we noted that the exposure of CD146^+^ cells to endothelial medium enrich with Met decreased this ratio (Fig. 5). Therefore, the application of autophagy modulators during differentiation of CD146^+^ cells toward different lineages such as EC, PC, and CM could alter the cellular fatty acid composition.

### Autophagy modulation altered the exosome biogenesis

**Fig. 6:**
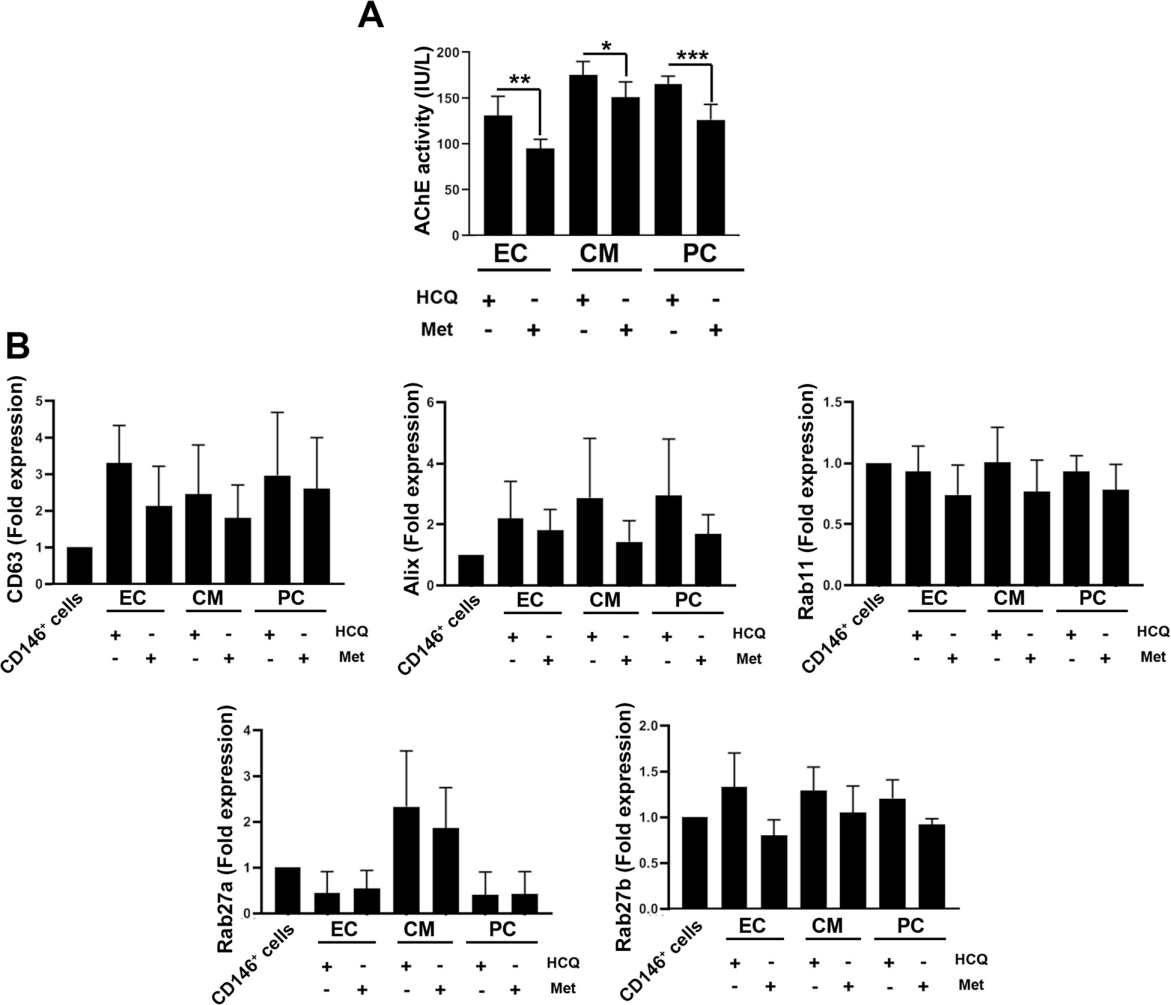
Exosome biogenesis assay (**a** and **b**). Measuring AChE in supernatant cell culture (**a**). Data showed that autophagy stimulation by Met decreased AChE activity compared to the HCQ groups. Real-time PCR analysis revealed the expression of CD63, Alix, Rab11, Rab27a, and Rab27b in stimulated autophagy groups compared to HCQ-treated cells (**b**; *n* = 3). These values did not reach significant levels. Student *t* test. *p < 0.05; **p < 0.01; ***p < 0.001

To further assess the modulation of autophagy on exosome biogenesis, AChE activity and the expression of genes including CD63, ALIX, Rab11, Rab27a, and Rab27b were measured. Data revealed the increase of exosome secretion to the supernatant in cells treated with HCQ compared to the Met groups (Fig. 6a). These data demonstrated that the treatment of CD146^+^ cells with autophagy blockers, 15-μM HCQ, increases exosome abscission and activates paracrine activity. A similar pattern was found in the expression of CD63, ALIX, Rab11, Rab27a, and Rab27b (Fig. 6b). Despite induction in molecular pathways in exosome biogenesis, localization, and abscission of HCQ-treated cells; however, these changes did not reach statistically significant changes.

## Discussion

The combinatorial cell therapy is touted as an intriguing approach to reach efficient results in terms of cardiac regeneration after myocardial infarction [26]. Therefore, the study of multiple differentiation capacity of distinct stem cells or progenitors could enable us in concomitant promotion of angiogenesis and cardiomyogenesis [27]. More than a decade, studies have suggested that different signaling pathways, such as autophagy, are involved to commence a novel cell-fate acquisition in different cell types [28]. Due to a lack of sufficient data, we reasoned that pharmacological modulation of autophagy (either stimulation or inhibition) in bone marrow-derived CD146^+^ cells would modulate the differentiation capacity and further change regenerative capacity. To this end, 72-h incubation of CD146^+^ cells with 15-μM HCQ revealed significant accumulation of intracellular vacuoles determined by LysoTracker staining, showing successful lysosomal dysfunction and autophagic inhibition [29]. In this study, we noted 72-h incubation of differentiating CD146^+^ cells with 50-μM Met-induced protein synthesis of Beclin-1, and an increase in LC3-II/LC3-I ratio compared to the HCQ-treated groups. In contrast to CD146^+^ cells committed to endothelial lineage, a concurrent decrease in p62 in cells from PC and CM groups showed the completion of autophagic response after the exposure to the Met, while the current results showed contradictory P62 changes after autophagy modulation in progenitor cells oriented toward endothelial lineage. One reason for this discrepancy would be that the treatment of cells with lineage-specific factors could alter or blunt the normal function of the autophagic signaling pathway effector [30]. Therefore, one could hypothesize that the treatment of human bone marrow CD146^+^ cells could alter cellular levels of main autophagic regulators such as Beclin-1, P62, and LC3II/I ratio. In support of this statement, the inhibition of the insulin growth factor receptor could perturb autophagic response by modulation of protein kinase C [31]. According to the existence of different growth factors in the differentiation medium, it is logical to suppose that modulation of autophagy-related factors varies depending on the cell phenotype and activation of signaling pathways.

Interestingly, analysis of nitrosative stress showed significantly higher NO levels in groups treated with autophagy inhibitor HCQ compared to the Met-treated CD146^+^ cells. According to the results from the MTT test and NO analysis, it seems that 72-h incubation of CD146^+^ cells with autophagy inhibitor committed to different lineages could reduce cell viability via the nitrosative stress [32]. Considering different levels of NO in EC, PC, and CM, it seems that progenitor cells respond differently to autophagy modulation upon differentiation to different lineages [33]. According to our result, the production of NO is induced in a condition enriched with Met compared to the HCQ group. According to data from real-time PCR analysis and NO assay, one could hypothesize that the induction of autophagy could trigger the differentiation capacity of CD146^+^ toward EC, PC, and CM in the genomic levels. The lack of significant changes in protein levels of lineage-specific factors may point to the necessity for prolonged incubation periods at the proteomic level. The differentiation capacity of CD146^+^ cells was also monitored after autophagy modulation. Real-time PCR analysis showed the potency of 50-μM Met to upregulate the mRNA expression of lineage-specific factors such as endothelial VE-cadherin, cardiac cTnI, and PC α-SMA compared to the control of CD146^+^ cells and HCQ-treated groups. Western blotting also exhibited an increase in protein levels of lineage-specific factors such as α-actinin, α-SMA, and vWF factor after modulation of autophagy compared to the control group. These changes were following the phosphorylation of Erk1/2 in which groups received Met and HCQ showed a more Erk1/2 phosphorylation rate compared to the control cells [34]. According to the previous data, the modulation of autophagic response, either stimulation or inhibition, could induce the same enzymatic activity in different levels [34, 35]. The increase of cellular lineage-specific factors in HCQ-treated cells in comparison with control CD146^+^ cells could be related to the abolition of protein clearance after autophagy flux inhibition. However, more mechanistic investigations are needed to address the importance of autophagic status on the cellular distribution of specific factors.

We found that simultaneous autophagy modulation of human bone marrow CD146^+^ cells committed to PC, EC, and CM lineages altered fatty acid profile in which an increased PUFA + MUFA/SFA ratio in Met-treated group was achieved compared to the HCQ counterpart. These data showed that the supplementation of differentiation culture medium with 50-μM Met shifted the differentiating CD146^+^ cell fatty acid profile from saturated to unsaturated status. Previous studies declared that the reduction of saturated fatty acids, notably palmitic acid, seems a strategic approach to increase autophagic flux [36]. This study showed the increase of MUFA and PUFA in differentiating CD146^+^ cells after autophagic stimulation.

To find the possible cross-talk between the RTKs and autophagy status, we measured the levels of VEGFR-1 and -2 and Tie-1 and -2. Despite a slight increase in the level of VEGFR-1 and -2 and Tie-1 and -2 in cells exposed to the Met, these values did not reach statistically significant levels. Further analysis of CD146^+^ cells after autophagy modulation showed a close interplay between autophagy and pro-inflammatory status [37]. We showed that the supplementation of differentiation media with 50-μM Met reduced inflammatory TNF-α and IL-6 production. Previous data confirmed that the promotion of autophagic response is a way to reduce pro-inflammatory status in the target cells [38]. The existence of the same pattern in the promotion of nitrosative status and inflammatory cytokine production could indirectly indicate that the inhibition of autophagy in progenitor cells could abort the survival rate after transplantation to the target sites [39]. There are controversies regarding the potency of inflammatory status on the differentiation capacity of stem cells [40, 41]. Liu et al. showed that prolonged pro-inflammatory status could abrogate the differentiation capacity of stem cells [40]. In contrast, Pourgholaminejad and co-workers highlighted the potency of cytokine in accelerating MSC differentiation capacity [41]. Although the incubation of CD146^+^ cells with Met decreased inflammation rate, this modulation did not alter differentiation capacity toward three lineages in vitro compared to the condition exposed to the HCQ. Another interesting data from this experiment was the potency of Met to alter the biosynthesis of MUFA and PUFA in CD146^+^ cells committed to the CM lineage but not EC and PC phenotypes. It seems that autophagy stimulation/inhibition could alter the synthesis of MUFA, PUFA, and SFA in a specific condition. Hurley and co-workers investigated that the addition of oleic acid, but not SFA such as palmitic acid, could promote the differentiation of rat skeletal muscle cells in vitro via engaging peroxisome proliferator-activated receptors alpha or gamma [42].

This study aimed also to define the interplay between the autophagy and paracrine activity in CD146^+^ cells by monitoring the exosome secretion capacity. Our data showed the exosome secretion capacity indicated by supernatant AChE activity and stimulation of different genes involved in exosomes synthesis, transfer, and abscission in HCQ-treated cells. It seems that the inhibition of autophagy in differentiating cells promotes paracrine activity in vitro [43]. Commensurate with data from the current experiment and previous studies, it seems that the inhibition of autophagy and fatty acid change could alter the paracrine ability of progenitor and differentiating cells and increase of PUFA and MUFA is efficient in the secretion of exosomes [44].

## Conclusion

In conclusion, it seems that the modulation of autophagy could alter the differentiation potential and exosome secretion of human CD146^+^ cells in vitro.

## Data Availability

The datasets used and/or analyzed during the current study are available from the corresponding author on reasonable request.

## Abbreviations

MTT: 3-(4,5-Dimethylthiazol-2-yl)-2,5-diphenyl tetrazolium bromide
DAPI: 4′,6-Diamidino-2-phenylindole
AChE: Acetylcholine esterase
α-SMA: Alpha-smooth muscle actin
Ang-1: Angiopoietin-1
cTnI: Cardiac troponin-I
CMs: Cardiomyocytes
CD146: Melanoma cell adhesion molecules
GC: Chromatography
ECs: Endothelial cells
ELISA: Enzyme-linked immunosorbent assay
FBS: Fetal bovine serum
GC: Gas chromatography
HCQ: Hydroxychloroquine
IL-6: Interleukin 6
DMEM/LG: Low-content glucose Dulbecco’s modified Eagle medium
MACS: Magnetic-activated cell sorting
Met: Metformin
MUFA: Monounsaturated fatty acid
MNCs: Mononuclear cells
PCs: Pericytes
PBS: Phosphate-buffered saline solution
PUFA: Polyunsaturated fatty acid
ROS: Reactive oxygen species
RT-PCR: Real-time PCR
RTKs: Receptor tyrosine kinases
SFA: Saturated fatty acid
TNF-α: Tumor necrosis factor alpha
Tie-1: Tyrosine-protein kinase receptor-1
Tie-2: Tyrosine-protein kinase receptor-2
VE-cadherin: Vascular endothelial—cadherin
VEGFR-1: Vascular epithelial growth factor receptor-1
VEGFR-2: Vascular epithelial growth factor receptor-2
